# Quantum-optical spectroscopy of a two-level system using an electrically driven micropillar laser as a resonant excitation source

**DOI:** 10.1038/s41377-018-0045-6

**Published:** 2018-07-25

**Authors:** Sören Kreinberg, Tomislav Grbešić, Max Strauß, Alexander Carmele, Monika Emmerling, Christian Schneider, Sven Höfling, Xavier Porte, Stephan Reitzenstein

**Affiliations:** 1 Institut für Festkörperphysik, Technische Universität Berlin, 10623 Berlin, Germany; 2 Institut für Theoretische Physik, Technische Universität Berlin, 10623 Berlin, Germany; 3 Technische Physik, Julius-Maximilians-Universität Würzburg, 97074 Würzburg, Germany; 4 SUPA, School of Physics and Astronomy, University of St Andrews, St Andrews, KY16 9SS UK

## Abstract

Two-level emitters are the main building blocks of photonic quantum technologies and are model systems for the exploration of quantum optics in the solid state. Most interesting is the strict resonant excitation of such emitters to control their occupation coherently and to generate close to ideal quantum light, which is of utmost importance for applications in photonic quantum technology. To date, the approaches and experiments in this field have been performed exclusively using bulky lasers, which hinders the application of resonantly driven two-level emitters in compact photonic quantum systems. Here we address this issue and present a concept for a compact resonantly driven single-photon source by performing quantum-optical spectroscopy of a two-level system using a compact high-*β* microlaser as the excitation source. The two-level system is based on a semiconductor quantum dot (QD), which is excited resonantly by a fiber-coupled electrically driven micropillar laser. We dress the excitonic state of the QD under continuous wave excitation, and trigger the emission of single photons with strong multi-photon suppression ($\, g^{(2)}(0) = 0.02$) and high photon indistinguishability (*V* = 57±9%) via pulsed resonant excitation at 156 MHz. These results clearly demonstrate the high potential of our resonant excitation scheme, which can pave the way for compact electrically driven quantum light sources with excellent quantum properties to enable the implementation of advanced quantum communication protocols.

## Microlasers: tiny pillars yield practically perfect quantum light

Sending encrypted quantum data over long distances is set to become more feasible using a low-cost system for generating photons one at a time. Repeating signals in a quantum network requires techniques for emitting single photons that preserve information such as polarization states. Stephan Reitzenstein from the Technische Universität Berlin, Germany, and co-workers report that bulky lasers used in typical quantum repeaters can be downsized using low-dimensional semiconductor nanostructures known as quantum dots. They fabricated micropillar structures, each containing aluminum–gallium–arsenic-based quantum dots as active medium, which produce coherent laser pulses when electrically stimulated. By directing the micropillar-driven pulses onto another quantum dot that resonates after absorbing laser light, the team triggered emission of high-quality, individual photons that may be beneficial for the implementation of long-distance quantum communication protocols.

## Introduction

The physics of two-level systems constitutes the basis for quantum optics and quantum cavity electrodynamics. It also has an important impact in the field of photonic quantum technologies, where it enables the secure exchange of information via single photons^1–4^ as well as efficient quantum computation with linear optics^5^. In particular, single photons are key resources for quantum key distribution using the BB84 protocol and for more advanced schemes, such as the quantum repeater concept for long-distance quantum communication. In such protocols and in quantum secure direct communication^6^, information is usually encoded in the polarization of the photon, and on-demand sources emitting single photons with high indistinguishability are of major importance for the implementation of these protocols. In this context, semiconductor quantum dots (QDs) are nearly ideal two-level systems and can act as triggered sources of single photons^7^, where specific material properties can even be used for the direct generation of linearly polarized photons^8^. To explore the physics of QDs and the quantum nature of emission, different excitation schemes have been developed, which include simple non-resonant electrical and optical excitation as well as more advanced schemes, such as wetting-layer or *p*-shell resonant excitation^9–14^. Most interesting is the strict resonant excitation of the fundamental QD transition leading to resonance fluorescence (RF)^15–20^. From an experimental point of view, strict resonant excitation is very demanding because it requires laser stray-light suppression by typically more than six orders of magnitude^16,21,22^. Nevertheless, the development of efficient suppression schemes and the availability of mode-hop-free tunable lasers have led to huge progress in this field, and RF has become an important experimental technique in quantum nanophotonics. For instance, strict resonant excitation has been used to study the subnatural linewidth from a single QD^23^ and to explore the non-resonant dot-cavity coupling in microcavity systems^24^. It is interesting to note that to date, the related experiments have only been performed using bulky and expensive laser systems.

In view of applications in quantum communications, strict resonant excitation of QDs is highly advantageous because it leads to the emission of single photons with excellent quantum properties in terms of multi-photon suppression and photon indistinguishability^25^. Both aspects are crucial for advanced quantum communication protocols based on entanglement distribution via Bell-state measurements^26,27^. In addition, to enable “real-world” applications, it is highly interesting to develop compact electrically driven quantum light sources. Unfortunately, standard excitation schemes based on carrier injection via a pin-diode design are intrinsically non-resonant, limiting the achievable degree of indistinguishability^28^. To overcome this issue, an advanced excitation concept has been developed using an electrically driven microlaser to excite a single QD in a nearby microcavity system^29^. In this concept, quasi-resonant *p*-shell excitation was demonstrated^30^, but strict resonant excitation has not yet been achieved. In a similar scheme, a light-emitting diode was used for the on-chip excitation of a single QD^31^.

In this article, we demonstrate a fully nanophotonic approach to resonantly drive a QD acting as a two-level system and to generate single photons with excellent multi-photon suppression and a high degree of photon indistinguishability. Our concept is based on an electrically driven QD micropillar laser that resonantly drives a single QD located in a planar microcavity. To resonantly excite a two-level system, we use a microlaser spectrally matched to a QD, where the temperature of the microlaser is used as a fine-tuning knob in resonance scans. The experiments are performed under continuous wave (CW) and pulsed excitation of the electrically driven microlaser to observe Mollow-triplet spectra and the triggered emission of single photons with a Hong-Ou-Mandel (HOM) visibility of 57%, respectively. Our results show the potential of high-*β* microlasers to act as excitation sources in quantum optics experiments and represent an important step toward the development of integrated quantum nanophotonic circuits relying on small-scale coherent light sources for resonant excitation of quantum emitters. This concept may lead to a significant cost reduction in quantum optics experiments when using microlasers instead of large laser systems as excitation sources. Even more interesting will be the application of low-threshold microlasers in integrated quantum circuits and compact quantum light sources, where they can resonantly trigger the emission of single photons and photon pairs as key resources for photonic quantum technology.

**Fig. 1:**
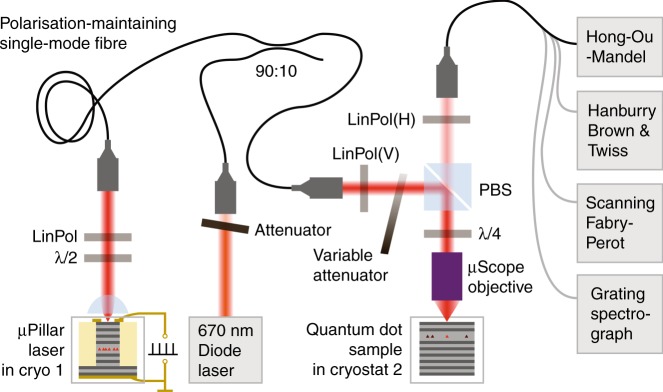
Schematic illustration of the experimental concept. Emission of the electrically driven microlaser in cryostat 1 is fiber-coupled to resonantly excite a single QD in cryostat 2. Applying either CW or pulsed excitation, dressing of the two-level system or triggered emission of single photons can be observed and verified by high-resolution spectroscopy and single-photon counting

Our experimental concept is illustrated in Fig. 1. It includes a QD micropillar laser located in cryostat 1 and a spectrally matched QD located in cryostat 2. The light emitted by the microlaser is coupled into a 10 m-long polarization-maintaining single-mode fiber, which is connected to the input port of the RF setup to excite the selected QD in cryostat 2. The microlaser is driven by an electrical voltage supply capable of delivering an adjustable DC bias and voltage pulses. The pulses have a width of 520 ps, an amplitude up to 8 V, and a maximum repetition rate of 312.5 MHz. Resonance tuning with the tuning range of approximately 35 GHz is enabled by changing the temperature of the microlaser in the 64–68 K range. The sample temperature of cryostat 2 is set to 7 K to minimize phonon-induced decoherence^32^ and carrier escape from the QDs in RF experiments. The need for cryogenic operation is also present for other relevant devices in quantum technology, such as superconducting nanowire single-photon detectors, and is in general required in advanced applications relying on quantum coherence, such as long-distance quantum communications networks. See the Materials and methods section for details on the sample technology and on the experimental setup.

For the planned quantum-optical studies, it is crucial to couple the emission of the high-*β* microlaser with sub-microwatt output power very efficiently into a single-mode fiber connecting the two cryostats. For this purpose, we collimate the microlaser emission via a single low-loss *f* = 20 mm aspheric lens in front of the optical window of cryostat 1. In this context, we would like to note that a slight deviation of the circular cross section splits the fundamental transverse micropillar mode into two gain-coupled mode components with a spectral splitting of 11 GHz. One of the two modes wins the gain competition and undergoes the lasing transition^33,34^. Emission of the lasing mode is selectively coupled into the polarization-maintaining single-mode fiber via a combination of a half-wave plate plus linear polarizer and a collimating beam coupler.

## Materials and methods

### Sample technology

The QD microlaser and the resonantly excited QD are based on AlGaAs heterostructures grown by molecular beam epitaxy. Both structures consist of high-quality AlAs/GaAs-based distributed Bragg reflectors (DBRs) forming a planar microcavity with a central one-*λ* GaAs cavity. A single layer of InGaAs QDs acts as the active medium. For the microlaser, the planar microcavity is composed of a rather high number (27 and 23) of mirror pairs in the *n*-doped (lower) and *p*-doped (upper) DBRs to ensure pronounced light–matter interaction and high-*β* lasing. A dense array of micropillar lasers with a diameter of 3 µm and a pitch of 60 µm are realized by high-resolution electron-beam lithography and subsequent reactive-ion etching. The sample is planarized with benzocyclobutene to mechanically support the ring-shaped upper Au contacts. This has the positive side effect of protecting the AlAs layers from oxidization. The realized array includes 62 electrically micropillar lasers emitting in the spectral range of 912–919 nm. We refer to ref.^35^ for further details on the fabrication of electrically contacted micropillars. The QD sample used in the RF experiments has a more asymmetric microcavity design with 24 and 5 mirror pairs in the lower and upper DBRs, promoting directional outcoupling of light with an extraction efficiency of up to 42%^36^. Due to the low QD density of 2 × 10^9^cm^−2^ and the presence of random photonic defects, this planar microcavity sample is highly suitable for single QD experiments and does not require lateral device processing. To address only a single QD with our laser spot, we have selected a sample position with a very low local QD density.

### Experimental setup

The experimental configuration consists of two independent helium-flow cryostats (cryostats 1 and 2, respectively), each placed on a different optical table. The electrically driven QD microlaser is installed in cryostat 1, and a single-mode fiber guides the laser light to the RF configuration in a cross-polarization configuration^18,19,21^ at cryostat 2. The resonant laser light enters the RF setup via a fiber beamsplitter, where it is superimposed with light from a low-power non-resonant support laser. The latter is a red diode laser (emission wavelength: 670 nm), the emission of which fills the charge traps adjacent to the QD to effectively gate the RF signal of the QD^37^. The combined lasers are collimated again to free space and are aligned with the RF detection beam path by a polarizing beamsplitter cube (PBS). Excitation of the QD and detection of RF is then performed confocally through a numerical aperture = 0.65, *f* = 4 mm microscope objective. The main purpose of the PBS is to strongly suppress the laser light reflected from the sample. To compensate for possible polarization ellipticity and to maximize laser stray-light suppression, a quarter-wave plate is placed in the excitation/detection path between the PBS and the microscope objective^19^. The detected light is fed into a polarization-maintaining single-mode fiber, both for spatial filtering and to facilitate quantum optics experiments. For Hanbury Brown and Twiss and HOM-style  single-photon correlation experiments, superconducting single-photon detectors with a time resolution full width at half maximum (FWHM) of 55 ps are correlated. High-resolution RF spectra are recorded using a scanning Fabry-Perot interferometer with a spectral resolution of 100 MHz.

## Results and discussion

In this work, we apply an electrically driven microlaser to demonstrate for the first time the high potential of micro- and nanolasers in quantum-optical spectroscopy. Indeed, while the research interest in miniaturized lasers has increased rapidly in recent years, their applicability as resonant excitation sources in quantum nanophotonics has been widely unexplored to date. To enable related studies under strict resonant excitation, it is crucial to obtain a microlaser that (a) can be operated electrically under CW and pulsed operation with an emission pulse width significantly shorter than the spontaneous emission lifetime (here 510 ps) of the QD, (b) shows single-mode emission with an emission linewidth significantly smaller than the homogeneous linewidth of approximately 1 GHz, (c) is spectrally matched with a target QD within the available temperature-tuning range on the order of 500 GHz, and (d) has sufficiently high optical output power of approximately 100–500 nW at the single-mode fiber output to at least saturate the QD transition.

**Fig. 2:**
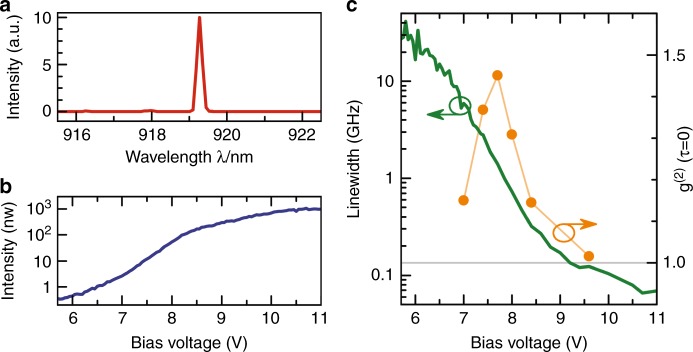
Characterization of the electrically driven micropillar laser under CW excitation. **a** EL spectrum of the QD micropillar laser showing clean emission of the fundamental mode. Higher-order lateral modes of the micropillar are well suppressed (see SI for details). **b** Input–output dependence of the electrically driven QD micropillar laser with a threshold pump voltage of approximately 7–8 V. **c** Equal-time second-order photon autocorrelation function (as measured) and spectral linewidth of the QD microlaser (deconvoluted taking the spectral resolution of the Fabry-Perot interferometer (0.1 GHz) into account). The nonlinear input–output characteristics in conjunction with the narrowing of the emission linewidth by more than three orders of magnitude and the transition of ${{g}}^{(2)}(0)$ from values larger than one to unity are clear indications of predominantly stimulated emission of the QD microlaser above the threshold

To meet these stringent requirements, we first performed reference measurements using a conventional tunable laser as the excitation source to select a QD showing pronounced and clean RF at 920 nm (see SI for more details on the reference measurement), where 920 nm corresponds to the central wavelength reachable by the micropillar lasers within the patterned array. All measurements shown in this paper are performed on this selected QD. In the second step, we chose a micropillar laser with a slightly shorter emission wavelength of 919 nm at 10 K so that it can be spectrally matched with the QD wavelength at 66 K. Figure 2a shows the 32 K electroluminescence emission spectrum of the microlaser at the output of the single-mode fiber. Without any spectral filtering, we observe clear single-mode emission with a side-mode suppression ratio of 19 dB and no significant contribution from GaAs or wetting-layer emission (see SI). Emission of the laser is coupled into a single-mode fiber, leading to the output power of 350 nW (at *V*_bias_ = 10.2 V) at the fiber output.

Figure 2b, c presents the corresponding voltage-dependent output power and spectral linewidth of the micropillar laser, respectively. The onset of laser action is indicated by the nonlinear increase of the output intensity between *V*_bias_ = 7 and 8 V accompanied by a strong decrease of the emission linewidth to values well below 0.1 GHz. The associated transition from predominantly spontaneous emission to stimulated emission is confirmed by measurements of the bias voltage-dependent second-order photon autocorrelation function $g^{(2)}(\tau )$, which shows the typical bunching behavior in the threshold region with $g^{(2)}(0) > 1$ and a transition toward coherent emission associated with $g^{(2)}(0) = 1$ at high excitation^38,39^. It is worth noting that the equal-time photon correlation only approaches $g^{(2)}(0) = 1$ when the linewidth is already reduced by a factor of 100, in accordance with ref.^40^. To avoid possible issues caused by the response time of the detectors, we attenuated the microlaser output in the $g^{(2)}(\tau )$ measurements above the threshold, keeping the count rate per detector always below 1 MHz.

**Fig. 3:**
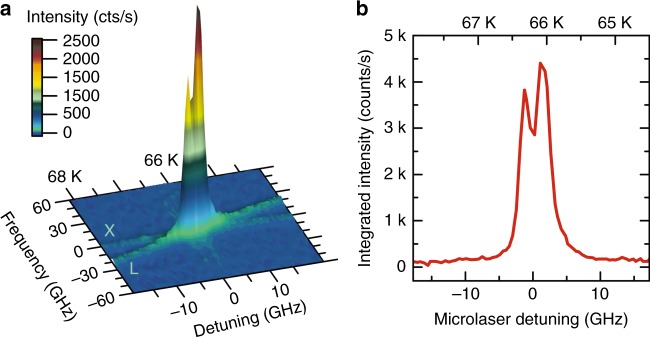
Resonance fluorescence (RF) of a single QD under CW microlaser excitation (*V*_bias_ = 10.2 V). **a** 3D surface plot of the QD emission intensity as a function of the frequency *f* and the microlaser detuning Δ. Using temperature tuning in the range of 64–68 K, the laser emission (L) is tuned through the spectral resonance of the selected QD transition (X). A strong RF signal is observed in resonance at approximately 66 K. **b** Emission intensity of the QD vs. laser detuning integrated over the spectral range of $- 60\,{\mathrm{GHz}} \le {{f}} \le 60\,{\mathrm{GHz}}$ displayed in (**a**). The double-peak structure is attributed to the fine-structure splitting of the excitonic transition

Having fulfilled the requirements (a)–(d) discussed above, we are prepared for RF experiments using the selected QD micropillar laser as a coherent excitation source. For this purpose, the temperature of the fiber-coupled microlaser in cryostat 1 is gradually varied between 64 and 68 K, and emission of the QD in cryostat 2 is recorded via the attached RF setup. The corresponding emission spectra (under CW excitation) are presented in Fig. 3a as a color-scale intensity map. While only weak emission of the QD and strongly suppressed laser emission can be detected under resonant conditions for QD-laser detuning >10 GHz, strong and very pronounced RF emission occurs at resonance. In fact, when scanning the microlaser emission over the QD *s*-shell resonance, a double-peak response with a splitting of 5 GHz detuning can be resolved (cf. Fig. 3b). This splitting is attributed to the fine-structure splitting of the excitonic transition of the QD^41^. The measurements presented in Fig. 3a, b also indicate that the reflected laser light and the QD emission due to the above-band excitation by the red laser make only marginal contributions to the RF signal. In the present approach, both the microlasers and the QDs are fabricated to emit at similar wavelengths. Nevertheless, the fine tuning of the wavelengths required for RF is still a very demanding prerequisite that we overcome by selecting the appropriate microlaser and QD among many candidates. A more deterministic approach will require deterministic nanofabrication of suitable QD-microcavity systems at the target wavelength^42^ and either electro-optical Stark tuning^43^ or more advanced strain-tuning^44^ for spectral fine tuning of the QD.

**Fig. 4:**
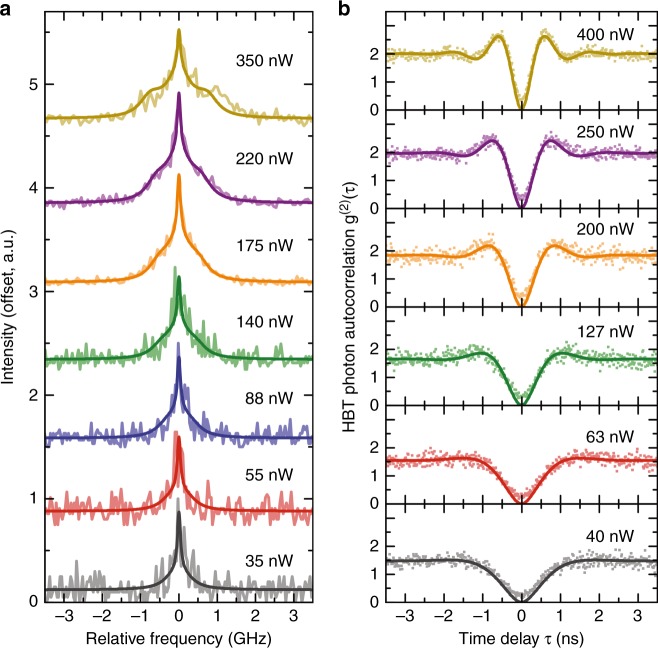
Excitation-dependent resonance fluorescence (RF) emission spectra and photon autocorrelation function under CW microlaser excitation. **a** High-resolution RF emission spectra for different excitation powers. With increasing excitation power, we observe a transition of the single emission line toward a Mollow-triplet-like emission spectrum. **b** Second-order photon autocorrelation function ${{g}}^{(2)}({{\tau }})$ of the resonantly driven QD. The strong antibunching at zero time delay ${{\tau }} = 0$ indicates single-photon emission. At higher laser powers, the narrowing of the antibunching peak together with the directly visible Rabi oscillations indicates coherent excitation of the two-level system

Coherent excitation of the QD-based two-level system is demonstrated in Fig. 4. Through the attenuation of the micropillar laser emission, we cover a CW excitation power range of 35–400 nW. Figure 4a shows the corresponding emission spectra recorded with a high-resolution Fabry-Perot scanning interferometer. With increasing excitation power, we observe the characteristic line broadening of the single emission line with a measured FWHM of 600 MHz at low drive toward the evolution of the Mollow triplet at high excitation strengths^45^. The splitting of the outer lines of the Mollow triplet with respect to the center amounts to 640 MHz at 350 nW. The occurrence of this important signature of coherent excitation is confirmed for the studied QD by reference measurements over a wider range of excitation powers using a standard tunable laser (see SI).

The quantum nature of RF emission is investigated by measuring the second-order photon autocorrelation function $g^{(2)}(\tau )$, again under CW excitation via the electrically driven microlaser. As seen in Fig. 4b, the excitation power-dependent photon correlation reveals pronounced antibunching statistics with strong suppression of multi-photon emission events associated with $g^{(2)}(0) < 0.4$. Upon increasing the excitation power from 40 to 400 nW, the simple antibunching dip evolves into a periodically modulated autocorrelation function. The observed signatures are associated with Rabi oscillations in agreement with the Mollow triplet observed in the frequency domain (cf. Fig. 4a). Importantly, we observe $g^{(2)}(\tau ) > 1$ in the vicinity of zero time delay $\tau \approx 0$. This photon bunching increases slightly with increasing excitation power and indicates the blinking of the QD due to metastable processes^46^.

**Fig. 5:**
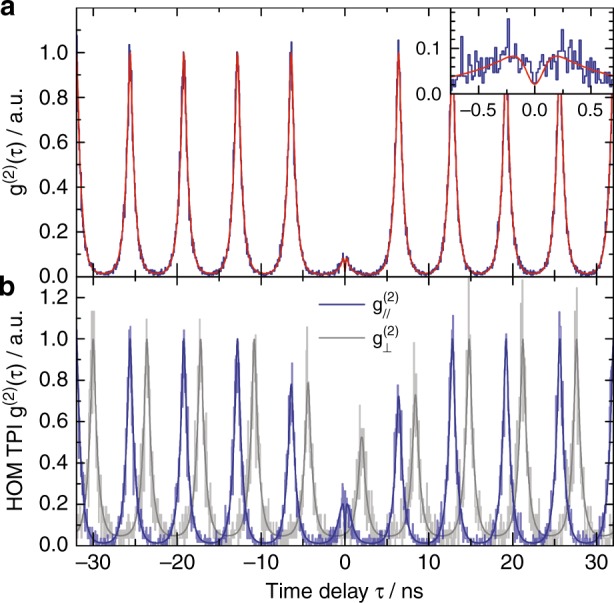
Demonstration of triggered single-photon emission and photon indistinguishability under pulsed microlaser excitation. **a** Second-order photon autocorrelation under pulsed resonant excitation of the QD (pulse area: $0.9{{\pi }}$). Triggered single-photon emission is clearly demonstrated by strong antibunching with ${{g}}^{(2)}(0) \ll 0.5$. The inset shows that the non-ideal ${{g}}^{(2)}(0)$ value can mainly be attributed to repeated QD excitation and decay within a single long-lasting laser pulse. This interpretation is confirmed by numeric modeling (red solid trace). **b** HOM histograms measured under co-polarized and cross-polarized (shifted by ${{\delta \tau }} = 2\,{\mathrm{ns}}$ for the sake of clarity) configurations. Taking into account the non-ideal ${{g}}^{(2)}(0)$ value of the data presented in (**a**), we determine the HOM visibility to be ${{V}}_{{\mathrm{pure}}} = 0.57(9)$

To obtain more detailed insight into the RF emission features and to theoretically describe the experimental data presented in Fig. 4 we consider the QD as a two-level system with a spontaneous emission lifetime *T*_1_, a dephasing time *T*_2_, and an excitation power-dependent Rabi frequency Ω. $T_1 = 510\,{\mathrm{ps}}$ and $\Omega /\sqrt P = 2\pi \times 1.33\,{\mathrm{THz}}\,{\mathrm{W}}^{ - 1/2}$ were determined via time-resolved experiments under pulsed micropillar laser excitation (cf. Fig. 5) and by investigating the excitation power-dependent autocorrelation using a standard tunable laser (see SI). Using the formulas introduced in the SI, we are able to model the experimental data under the variation of *T*_2_. All optimal values of *T*_2_ lie in the vicinity of 500 ps. Assuming *T*_2_ = 500 ps, we obtain excellent quantitative agreement between experiment and theory, as seen in Fig. 4a, b, where solid lines present the calculated data from formulas S3 and S4, respectively.

For applications in photonic quantum technology, it is crucial to demonstrate the triggered emission of single photons with excellent quantum properties. For this purpose, we biased the microlaser with $V_{{\mathrm{bias}}} = 4.71\,{\mathrm{V}}$ below the onset of lasing and superimposed voltage pulses with $V_{{\mathrm{pp}}} = 8\,{\mathrm{V}}$, a width of 520 ps, and a repetition period of 6.4 ns. It is interesting to note that due to the nonlinear input–output dependence of the microlaser, the resulting optical emission pulses were shortened significantly to a width of 200 ps (FWHM). The ratio of the peak laser intensity to the strongest after-pulsing intensity was >18 dB (see SI). The pulsed emission was again coupled via the single-mode fiber in an RF configuration into cryostat 2 to resonantly excite the selected QD. The corresponding photon autocorrelation function was recorded at an excitation power of 22 nW and is presented in Fig. 5a. Pulsed emission of light is clearly identified by the train of correlation pulses separated by 6.4 ns, and triggered single-photon emission is evidenced by the strongly reduced peak at zero delay with $g^{(2)}(0) = 2\%$. The zoom-in presentation of $g^{(2)}(\tau )$ in the inset of Fig. 5a shows a characteristic substructure of the central $g^{(2)}(\tau )$ peak with a minimum at $\tau = 0$ and side peaks at a finite delay. This correlation feature indicates that the non-ideal multi-photon suppression is mainly due to repeated QD excitation and decay within a single long-lasting laser pulse. Numerical modeling (red solid trace, cf. SI for details) was used to confirm the nature of the central correlation feature and to extract the lifetime of $T_1 = 510\,{\mathrm{ps}}$ and the pulse area of 0.9*π* by fitting the modeled curve to the experimental data. Indeed, it was predicted that both increased pulse length^47^ and increased pulse area^48^ increase the probability of multi-photon-photon events. Thus, in the future, even better multi-photon suppression may be achieved by applying shorter electrical pulses to the microlaser.

Finally, we study the photon indistinguishability of emission under pulsed microlaser excitation via a fiber-coupled HOM two-photon interferometer with adjustable delay^32,49^. Here the delay of the associated Mach-Zehnder interferometer was matched to the pulse repetition rate of 6.4 ns of the electrical voltage source used to drive the microlaser. The resulting photon correlation diagram of emission from the resonantly excited QD is displayed in Fig. 5b, both in the co-polarized and cross-polarized measurement configurations. The experimental data are displayed as light blue and light gray lines, and the numerically modeled fitting data (see SI) are displayed as dark blue and dark gray lines. The only fitting parameter (except for background counts and scaling) is the imbalance of the second beamsplitter, which is the splitter at which the HOM effect occurs, which is found to be 8:9 and results in the different heights of the peaks at $\pm 6.4\,{\mathrm{ns}}$. A significant degree of photon indistinguishability is evidenced by strongly reduced coincidences in the co-polarized case, while for the cross-polarized case, we observe $g_ \bot ^{(2)}(0) \approx 0.5$, as expected for distinguishable photons. To determine the resulting two-photon interference visibility *V*, we first integrate the areas $A_n^{^{\parallel , \bot }}$ of the peaks centered at time delays $\tau = n \times 6.4\,{\mathrm{ns}},\,n \in \left\{ { - 7, - 6, - 5, \ldots ,6,7} \right\}$ for each polarization configuration. Then, using

1 $$\begin{array}{l}A_{{\mathrm{ref}}}^{\parallel , \bot } = \frac{1}{{12}}\mathop {\sum }\limits_{n = 2}^7 A_n^{\parallel , \bot } + A_{ - n}^{\parallel , \bot }\\ V = 1 - \frac{{A_0^\parallel A_{{\mathrm{ref}}}^ \bot }}{{A_{{\mathrm{ref}}}^\parallel A_0^ \bot }}\end{array}$$

we extract the raw two-photon interference visibility of $V = 0.44(4)$. When compensating for the non-zero $g^{(2)}(0)$ and for the slight HOM beamsplitter imbalance of 8:9, we obtain a two-photon visibility as high as $V_{{\mathrm{pure}}} = 0.57(9)$ (see SI for details). This value is higher than the 41% reported in ref.^28^ for the direct non-resonant electrical excitation of a QD via carrier injection in a pin-diode design. It is, however, significantly lower than the values exceeding 90% achieved by resonant excitation via standard mode-locked lasers with ps-pulse widths. Several possible effects can be considered in order to explain the non-ideal degree of photon indistinguishability, such as temperature-induced dephasing^32,50^ or spectral fluctuations^32^. In the present case, i.e., under resonant excitation at low temperature, we can exclude these effects. Instead, we attribute the reduced HOM visibility mainly to the rather long optical pulse width of 200 ps and to the non-Fourier-limited dephasing time $T_2 = 0.5\,{\mathrm{ns}} \approx T_1 < 2T_1$ (see Fig. S9). The increased laser pulse width in combination with strong pulse power ($0.9\pi$) leads to two-photon fluorescence pulses and in turn results in reduced HOM visibility^47,48,51^. On the other hand, the non-Fourier-limited *T*_2_ directly makes the photons more distinguishable either due to random phase changes or due to fine-structure splitting^11,52^ of the QD transition, thereby implying wavelength distinguishability. We therefore expect a strong improvement of the photon indistinguishability by carefully adjusting the detected polarization to a single QD transition only and by reducing the optical pulse length in future studies.

## Conclusion

In conclusion, we demonstrated a fully nanophotonic concept for the control of single-photon emission of a solid-state two-level system. The concept involves an electrically driven high-*β* microlaser that resonantly drives a semiconductor QD acting as a two-level system. This work demonstrates for the first time the applicability of micro- and nanolasers in advanced quantum optics experiments under strict resonant excitation. Temperature-induced spectral fine tuning of a suitable QD microlaser allows us to observe the dressing of the fundamental QD transition and the occurrence of Rabi oscillations in photon correlation measurements. Pulsed electrical excitation of the microlaser leads to the emission of single photons with high multi-photon suppression ($g^{(2)}(0) = 2\%$) and a HOM visibility as high as 57%. As such, our results show the great potential of combining and coupling nanophotonic devices to systems with enhanced functionality. In the future, our concept could be further developed into a fully integrated on-chip resonantly pumped quantum light sources with many interesting applications in photonic quantum information technology. For instance, the implementation of quantum repeater networks will strongly benefit from sources of single and indistinguishable photons resonantly triggered by integrated microlasers instead of the use of standard large-scale laser systems as excitation sources.

## Electronic supplementary material

Supplementary Information
