# Supplementary material for: Quantum-optical spectroscopy of a two-level system using an electrically driven micropillar laser as a resonant excitation source
Source: Light Sci Appl. 2018 Jul 25;7:41. doi: 10.1038/s41377-018-0045-6 (PMC6107011; doi:10.1038/s41377-018-0045-6)
Supplement: Supplementary file 1 — Supplementary Information [file 41377_2018_45_MOESM1_ESM.pdf]

Supplementary Information
Quantum-optical spectroscopy of a two-level system using an electrically driven micropillar laser as a resonant excitation source

Sören Kreinberg,[1] Tomislav Grbešić,[1] Max Strauß,[1] Alexander Carmele,[2] Monika Emmerling,[3] Christian Schneider,[3] Sven Höfling,[3,4] Xavier Porte,[1] and Stephan Reitzenstein[1]

[1] *Institut für Festkörperphysik, Technische Universität Berlin, 10623 Berlin, Germany*
[2] *Institut für Theoretische Physik, Technische Universität Berlin, 10623 Berlin, Germany*
[3] *Technische Physik, Julius-Maximilians-Universität Würzburg, 97074 Würzburg, Germany*
[4] *SUPA, School of Physics and Astronomy, University of St Andrews, St Andrews, KY16 9SS, United Kingdom*

SI. Spectral characteristics of the electrically driven and fiber-coupled micropillar laser

For the resonant excitation scheme applied in this work clean single-mode emission of the microlaser is crucial. To complement the narrow-band µEL spectrum shown in Fig. 2a we present an emission spectrum in a wider spectral range in Fig. S1. We would like to note that both spectra were obtained at the output of the single-mode fiber without spectral filtering. The presented continous wave (CW) µEL spectrum of the micropillar laser is dominated by emission of the fundamental laser mode and the laser light can be used directly to resonantly drive the QD.

SII. Resonant excitation at high excitation powers using a standard tunable laser

In order to investigate the coherent excitation of the QD (see Fig. 4a) under in a wider excitation power range not accessible with the microlaser, we performed additional measurements using a standard tunable laser. Fig. S2 shows the corresponding resonance fluorescence spectra at five different excitation powers between 17 µW and 6.75 mW. The Mollow-triplet like spectra are very well resolved in this high excitation regime and confirm the interpretation of the data presented in Fig. 4a.

The solid lines are calculated spectra according to Eq. S3. Fig. S3 additionally displays the excitation-power dependent resonance fluorescence in a 2D contour plot. From the data presented in this figure we determine the relation $\frac{\Omega}{\sqrt{P}} = 1.33 \cdot 2\pi\,\mathrm{THzW}^{-\frac{1}{2}}$.

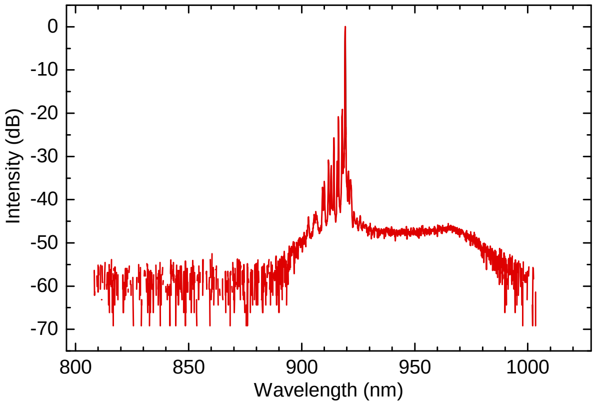

Figure S1. Overview µEL spectrum of the micropillar laser under CW excitation. The spectrum is recorded at the output of the single-mode fiber and shows pronounced single-mode emission of the fundamental laser mode. We would like to point out that no significant contribution from GaAs bandgap or wetting layer emission is detected.

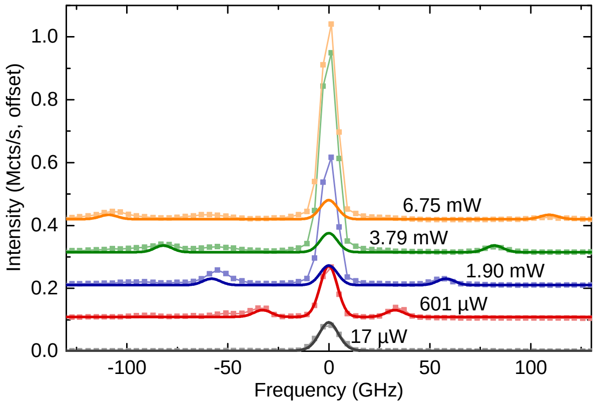

Figure S2. Resonance fluorescence spectra of the QD studied in the main text for tunable-laser excitation with five different excitation powers from 17 µW to 6.75 mW. The sidebands of the characteristic excitation power dependent splitting of the Mollow-triplet is clearly observed. Symbols present measured spectra and solid curves correspond to calculated spectra obtained by fitting the experimental data according to Eq. S3. A constant offset is introduced for the sake of clarity.

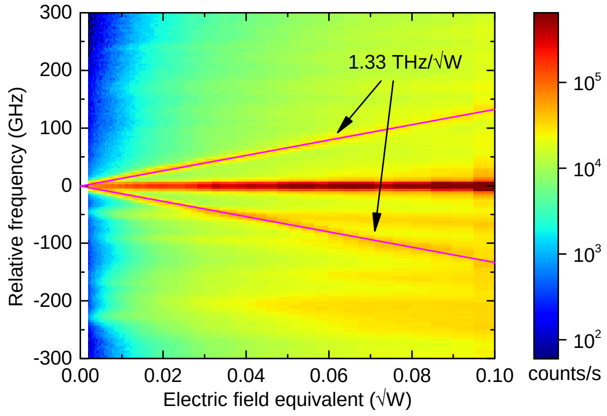

Figure S3. 2D contour plot of excitation power dependent resonance fluorescence spectra. The frequency distance of the the Mollow triplet sidebands to the carrier frequency divided by the square root of the excitation power is $1.33\,\mathrm{THz}/\sqrt{\mathrm{W}}$.

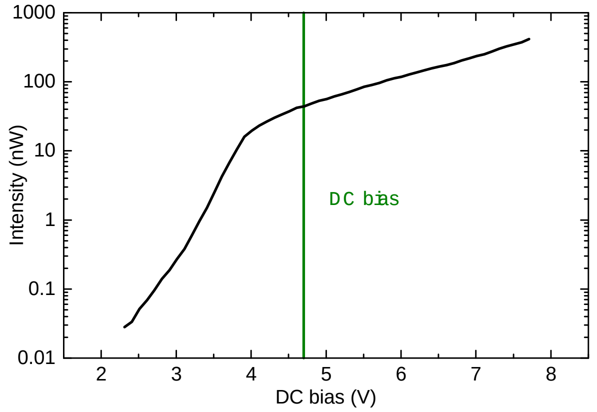

Figure S4. µPL intensity of micropillar laser in pulsed operation as a function of V_{bias} and $V_{\mathrm{peak}} = 8$ V for an electrical pulse-width of 520 ps and a pulse separation of 6.4 ns. The green line indicates the chosen DC bias ($V_{\mathrm{bias}} = 4.7\,\mathrm{V}$) chosen for the auto-correlation and HOM-measurements presented in Fig. 5 of the main text.

SIII. Emission properties of the micropillar laser under pulsed electrical excitation

As mentioned in the main part of the manuscript, single photons were triggered by exciting the QD with short resonant micropillar laser pulses. To generate these laser pulses, the supply voltage of the micropillar laser triggered electrically. In the applied excitation scheme the laser was biased with $V_{\mathrm{bias}} = 4.7$ V on top of which pulses with a peak voltage of $V_{\mathrm{peak}} = 8$ V and width of 520 ps FWHM were added. The pulse period is 6.4 ns corresponding to a repetition frequency of 156 MHz. Fig. S4 presents the output intensity of the microlaser as a function of the DC bias for constant pulse hight of 8 V. Fig. S5 displays the time resolved emission intensity of the microlaser in logarithmic scale and Fig. S6 shows the second-order auto-correlation function $g^{(2)}(\tau)$ of the micropillar, both $V_{\mathrm{peak}} = 8$ V. As expect for coherent laser emission $g^{(2)}(0) = 1.016(12)$.

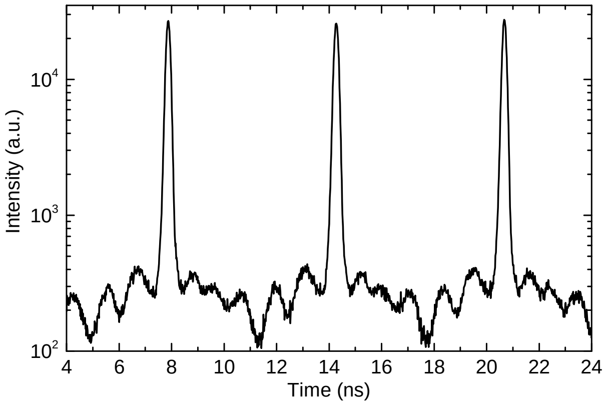

Figure S5. Time resolved µEL emission intensity of the pulsed microlaser. We observe low background signal with a ratio of signal to afterpulses and reflections of ≈ 75.

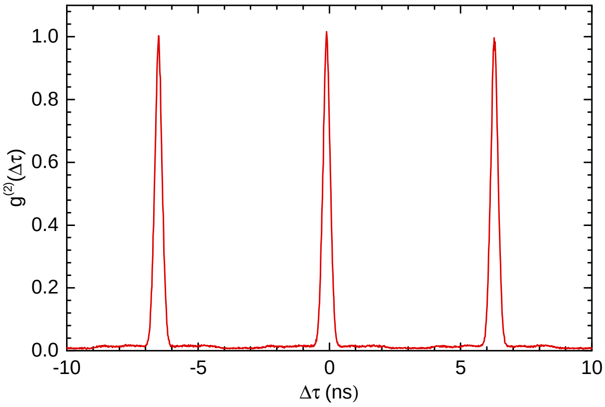

Figure S6. Second-order photon auto-correlation function $g^{(2)}(\tau)$ of the pulsed driven QD micropillar laser. From the data we extract $g^{(2)}(0) = 1.016(12)$ indicating coherent laser emission.

SIV. Theoretical description of resonance fluorescence

we treat the QD as a two-level system with the ground state $|g\rangle$ and the exited state $|e\rangle$, driven by a laser light-field oscillating with the angular frequency ω_{L} with the time-dependent envelope of arbitrary phase $\Omega(t)$. For resonant

driving the system can be described via the rotating wave approximation and in the rotating frame of ω_L by the Hamiltonian

$$H = \frac{\hbar}{2}\left[\Omega^*(t)\sigma_+ + \Omega(t)\sigma_-\right].$$

with the annihilation and creation operators $\sigma_- = |g\rangle\langle e|$ and $\sigma_+ = |e\rangle\langle g|$. The numerical simulation relies on the quantum-optical master equation in the Lindblad form[1]

$$\partial_t\,\rho(t) = -\frac{i}{\hbar}\left[H(t),\,\rho(t)\right] + \sum_\alpha L[C_\alpha]\rho(t)$$
$$L[C]\rho = C\rho C^\dagger - \frac{1}{2}C^\dagger C\rho - \frac{1}{2}\rho C^\dagger C$$

where the individual collapse operators C_α are: $C_{|g\rangle\langle e|} = \sqrt{\Gamma}\sigma_-$, $C_{|g\rangle\langle g|} = \sqrt{\gamma}\sigma_-\sigma_+$ and $C_{|e\rangle\langle e|} = \sqrt{\gamma}\sigma_+\sigma_-$. With the decay rate Γ given by the inverse of the excitonic lifetime T_1 and the pure dephasing rate γ given by $\gamma = \frac{1}{T_2} - \frac{\Gamma}{2}$.

SV. Theoretical description of the Hong-Ou-Mandel experiment under pulsed resonant excitation

In order to describe the experimental data presented in Fig. 5 of the main manuscript we use the master equation introduced in Sec. SIV. The envelope $\Omega(t)$ of the driving laser light field is given by a pulse train of Gaussian pulses with pulse separation $\delta = 6.4\,\mathrm{ns}$. Each pulse is of the FWHM w and covers a pulse area A:

$$\Omega(t) = \sum_k \frac{A}{\sqrt{2\pi}s}\exp\left(-\frac{t-k\delta}{2s^2}\right), \qquad w = 2\sqrt{2\ln 2}s$$

Following Fischer et al.,[2] we apply the quantum regression theorem to numerically calculate the correlation functions $G^{(1)}(t,\tau) = \langle\sigma_+(t)\sigma_-(t+\tau)\rangle$ and $G^{(2)}(\tau) = \int\langle\sigma_+(t)\sigma_+(t+\tau)\sigma_-(t+\tau)\sigma_-(t)\rangle\,\mathrm{d}t$. The second-order autocorrelation function $g^{(2)}(\tau)$ is proportional to $G^{(2)}(\tau)$ and since in experimental practice often no strict difference is made between $g^{(2)}$ and $G^{(2)}$, the calculation of $G^{(2)}(\tau)$ is sufficient.

For measuring the quantum-mechanical two-photon interference of two successive photons originating from the same source, a Mach-Zender interferometer is placed before the actual Hong-Ou-Mandel beam splitter.[3] The cross-correlogram of this experiment in cross- and co-polarized configuration is calculated similar to[4] but additionally for arbitrary beam-splitter transmission / reflection ratios, yielding

$$G^{(2)}_\perp = (\Theta_1^2 + R_1^2)\Theta_2 R_2 G^{(2)}(\tau) + \Theta_1 R_1 \Theta_2^2 G^{(2)}(\tau-\Delta) + \Theta_1 R_1 R_2^2 G^{(2)}(\tau+\Delta)$$
$$G^{(2)}_\parallel = G^{(2)}_\perp - 2\Theta_1 R_1 \Theta_2 R_2 \int G^{(1)}(t-\Delta,\tau)^* G^{(1)}(t,\tau)\mathrm{d}t$$

where $\Theta_{1/2}$ and $R_{1/2}$ are the transmitivity and reflectivity of the first / second beamsplitter and Δ is the delay corresponding to the arm-length difference of the Mach-Zehnder interferometer.

SVI. Theoretical description of resonance fluorescence under CW excitation

The quantum optical master equation (see Sec. SIV) leads to the Bloch equation, which - in case of CW excitation $\Omega(t) = \Omega$ - can be cast conviently in the following form:

$$\begin{aligned}\partial_t P(t) &= -\Gamma_P P(t) - i|\Omega|^2 W(t)\\ \partial_t W(t) &= -\Gamma_R (W(t)+1) - iP(t),\end{aligned} \tag{S1}$$

with the abbreviations:

$$P(t) = \langle\sigma_+(t) - \sigma_-(t)\rangle\,, \qquad W(t) = \langle\sigma_z(t)\rangle\,, \qquad \left\langle\sigma_z^2(t)\right\rangle = 1$$
$$\sigma_z = |e\rangle\langle e| - |b\rangle\langle b|\,, \qquad \Gamma_\mathrm{R} = \Gamma = \frac{1}{T_1}, \qquad \Gamma_\mathrm{P} = \frac{1}{T_2} = \frac{\Gamma}{2} + \gamma$$

With the inversion operator σ_z. Now, it is straight forward to solve these differential equations via Laplace transformation or matrix diagonalization.

Power spectrum and Mollow triplet

For deriving the theoretical formula of the power spectrum, predicting the Mollow-triplet presented in Fig. 4, the first-order correlation function needs to be computed:

$$S(\omega) = \lim_{t\to\infty} \int_0^\infty d\tau \, \langle \sigma_+(t)\sigma_-(t+\tau)\rangle \, e^{i\omega\tau}, \tag{S2}$$

so the time-dynamics of the expectation value of transition operator is necessary, which reads

$$\begin{aligned}\langle\sigma_-(t)\rangle = & -i\Omega\frac{A_3}{\Gamma_\mathrm{P}} + e^{-\Gamma_\mathrm{P} t}\left(\langle\sigma_-(0)\rangle + i\Omega\frac{A_3}{\Gamma_\mathrm{P}} - i\Omega\frac{A_1\Gamma_- + A_2\Omega_\mathrm{R}}{\Gamma_-^2+\Omega_\mathrm{R}^2}\right)\\ & - i\Omega e^{-\Gamma_+ t}\left(\frac{\Omega_\mathrm{R}A_1 - \Gamma_- A_2}{\Gamma_-^2+\Omega_\mathrm{R}^2}\sin(\Omega_\mathrm{R}t) - \frac{A_1\Gamma_- + A_2\Omega_\mathrm{R}}{\Gamma_-^2+\Omega_\mathrm{R}^2}\cos(\Omega_\mathrm{R}t)\right)\end{aligned}$$

using following abbreviations

$$\begin{aligned}\Gamma_\pm &= \frac{\Gamma_\mathrm{R}\pm\Gamma_\mathrm{P}}{2}, \qquad \Omega_\mathrm{R}^2 = \Omega^2 - \Gamma_-^2, \qquad A_1 = \langle\sigma_z(0)\rangle + \frac{\Gamma_\mathrm{R}\Gamma_\mathrm{P}\left\langle\sigma_z^2(0)\right\rangle}{\Omega_\mathrm{R}^2+\Gamma_+^2}\\ A_3 &= -\frac{\Gamma_\mathrm{R}\Gamma_\mathrm{P}\left\langle\sigma_z^2(0)\right\rangle}{\Omega_\mathrm{R}^2+\Gamma_+^2} \qquad A_2 = \frac{4\Omega}{\Omega_\mathrm{R}}\mathrm{Im}\left[\langle\sigma_-(0)\rangle\right] - \frac{\Gamma_-}{\Omega_\mathrm{R}}\langle\sigma_z(0)\rangle - \frac{\Gamma_\mathrm{R}}{\Omega_\mathrm{R}}\frac{\Omega_\mathrm{R}^2+\Gamma_+\Gamma_-}{\Omega_\mathrm{R}^2+\Gamma_+^2}\left\langle\sigma_z^2(0)\right\rangle.\end{aligned}$$

For all possible initial conditions, the solution allow the calculation of all superposition of the exciton dynamics and therefore also the two-time correlation function in the steady-state limit: $\lim_{t\to\infty}\langle\sigma_+(t)\sigma_-(t+\tau)\rangle = \langle\sigma_+(0)\sigma_-(\tau)\rangle_\infty$. Therefore, the steady state values of the system are needed: $\chi = \langle\sigma_+\rangle_\infty = -i\Omega A_3/\Gamma_\mathrm{P}$ and $\langle\sigma_z\rangle_\infty = A_3$. Via the quantum regression theorem, the dynamics of the polarization dynamics is given with different initial values, such as $A_3(\sigma_+) = \langle\sigma_+(0)\sigma_z(0)\rangle - A_1(\sigma_+)$. This leads to the following correlation function dynamics:

$$\begin{aligned}\langle\sigma_+(0)\sigma_-(\tau)\rangle_\infty = & i\frac{\Omega\chi}{\Gamma_\mathrm{R}}e^{-\Gamma_\mathrm{P}\tau} + i\frac{\Omega\chi}{\Omega_\mathrm{R}}e^{-\Gamma_+\tau}\sin(\Omega_\mathrm{R}\tau)\left(\frac{\Gamma_-}{\Gamma_\mathrm{R}} + 1 - \frac{\Gamma_+\Gamma_\mathrm{R}}{4\Omega^2+\Gamma_\mathrm{R}\Gamma_\mathrm{P}}\right)\\ & + i\frac{\Omega\chi}{\Gamma_\mathrm{R}}e^{-\Gamma_+\tau}\cos(\Omega_\mathrm{R}\tau)\left(1 - \frac{\Gamma_\mathrm{R}^2}{4\Omega^2+\Gamma_\mathrm{R}\Gamma_\mathrm{P}}\right) + i\frac{\Omega\chi}{\Gamma_\mathrm{R}}\frac{\Gamma_\mathrm{R}^2}{4\Omega^2+\Gamma_\mathrm{R}\Gamma_\mathrm{P}}.\end{aligned}$$

Now, given this dynamics, the spectrum can easily be calculated, and the formula in the main text is derived with the abbreviation:

$$\langle\sigma_+(0)\sigma_-(\tau)\rangle_\infty = i\frac{\Omega\chi}{\Gamma_\mathrm{R}}\left(e^{-\Gamma_\mathrm{P}\tau} + \frac{\Gamma_\mathrm{R}^2}{4\Omega^2+\Gamma_\mathrm{R}\Gamma_\mathrm{P}}\right) + e^{-\Gamma_+\tau}\sin(\Omega_\mathrm{R}\tau)Z_S + e^{-\Gamma_+\tau}\cos(\Omega_\mathrm{R}\tau)Z_C.$$

Changing back from the rotating frame to the laboratory frame and applying the single-sided Fourier transform Eq. S2, the result for the power spectrum presented in Fig. 4 is given by

$$\begin{aligned}S(\omega) = & \frac{\Gamma_\mathrm{R}^2\pi}{\Omega^2+\Gamma_\mathrm{P}\Gamma_\mathrm{R}}\delta(\omega-\omega_0) + \frac{\Gamma_\mathrm{P}}{(\omega-\omega_0)^2+\Gamma_\mathrm{P}^2} + \frac{1}{2}\frac{\Gamma_+Z_\mathrm{C} + (\omega-\omega_0+\Omega_\mathrm{R})Z_\mathrm{S}}{\Gamma_+^2 + (\omega-\omega_0+\Omega_\mathrm{R})^2} + \frac{1}{2}\frac{\Gamma_+Z_\mathrm{C} - (\omega-\omega_0-\Omega_\mathrm{R})Z_\mathrm{S}}{\Gamma_+^2 + (\omega-\omega_0-\Omega_\mathrm{R})^2}, \\ Z_\mathrm{C} = & 1 - \frac{\Gamma_\mathrm{R}^2}{4\Omega^2+\Gamma_\mathrm{P}\Gamma_\mathrm{R}}, \qquad Z_\mathrm{S} = \frac{1}{\Omega_\mathrm{R}}\left(\Gamma_- + \Gamma_+ - \frac{\Gamma_+\Gamma_\mathrm{R}}{\Omega^2+\Gamma_\mathrm{P}\Gamma_\mathrm{R}}\right).\end{aligned} \tag{S3}$$

Two-photon autocorrelation function $g^{(2)}(\tau)$

The theoretical description of the photon-autocorrelation function was derived straight-forward from Eq. S1 by diagonalization and subsequent application of the quantum regression theorem. The result is in agreement with the ansatz published in Ref.[5] and is given by

$$g^{(2)}(\tau) = B\left[1 - \mathrm{e}^{-\Gamma_+|\tau|}\left(\cos(\Omega_\mathrm{R}|\tau|) + \frac{\Gamma_+}{\Omega_\mathrm{R}}\sin(\Omega_\mathrm{R}|\tau|)\right)\right], \quad \tau\in[-4\,\mathrm{ns}, 4\,\mathrm{ns}] \tag{S4}$$

Excitation power (nW)	$g^{(2)}(0)$	B	$\tilde{g}^{(2)}(0)$
400	0.37	2.01	0.18
250	0.36	1.97	0.18
200	0.37	1.84	0.20
127	0.32	1.65	0.19
63	0.28	1.55	0.18
40	0.26	1.47	0.18

Table I. Equal-time two-photon autocorrelation function without ($g^{(2)}(0)$) and with ($\tilde{g}^{(2)}(0)$) blinking compensation and blinking-factor B. Due to blinking of the QD, the $g^{(2)}(\tau)$ is increased by a factor B for small time delays τ. Hence, the blinking-compensated autocorrelation is calculated by normalizing the original function to 1 in the vicinity of zero time delay: $\tilde{g}^{(2)}(0) = g^{(2)}(0)/B$.

where an excitation power dependent factor $B > 1$ accounts for blinking of the QD. Only in the short-delay limit, B can be approximated to be constant, thus the limitation of the domain to $\tau \in [-4\,\mathrm{ns}, 4\,\mathrm{ns}]$. For longer time delays, B is expected to decrease and eventually converge to $B = 1$ in the infinite-delay limit. Table I lists the equal-time two-photon autocorrelation functions without ($g^{(2)}(0)$) and with ($\tilde{g}^{(2)}(0)$) blinking compensation as well as the blinking-factor B.

SVII. Hong-Ou-Mandel visibility analysis, compensating non-vanishing $g^{(2)}(\tau = 0)$

In a Hong-Ou-Mandel (HOM) style two-photon quantum-interference experiment, multi-photon pulses entering the beamsplitter lead to an increase of equal-time coincidences, even if the photons are indistinguishable. Thus an $g^{(2)}(\tau = 0) > 0$ masks the quantum-interference and, if not taken into account properly, leads to an underestimation of the photon indistinguishability in terms of the visibility V (c.f. Eq. 1 in the main manuscript). In order to extract the pure HOM-visibility V_{pure} from the experimental data presented in Fig. 5b we follow the procedure described in Ref..[6] Considering our experimental HOM-configuration presented in Fig. 1 of the main text with a pulse-separation of δ (pulse rate: $\frac{1}{\delta}$), we obtain peak-areas A_i (while neglecting a proportionality constant w.r.t. the total detected counts) for the coincidence peaks:

$$A_0 = 2g^{(2)}(0)(T_1^2 + R_1^2)R_2T_2 + (g^{(2)}(\delta) + g^{(2)}(-\delta))R_1T_1(R_2^2 + T_2^2)(1 - V)$$

$$A_\delta = 2g^{(2)}(0)T_1R_1R_2^2 + 2(T_1^2 + R_1^2)R_2T_2 + 2T_1R_1T_2^2$$

$$A_{-\delta} = 2g^{(2)}(0)T_1R_1T_2^2 + 2(T_1^2 + R_1^2)R_2T_2 + 2T_1R_1R_2^2$$

$$A_{2\delta} = A_{-2\delta} = 2T_1R_1(T_2^2 + R_2^2) + 2(T_1^2 + R_1^2)R_2T_2$$

We further define:

$$N = A_{2\delta} + A_{-2\delta} - A_\delta - A_{-\delta} = 2T_1R_1(T_2^2 + R_2^2)(1 - g^{(2)}(0))$$

and:

$$A_{\text{NM}}^{\parallel/\perp} = \frac{A_\delta^{\parallel/\perp} + A_{-\delta}^{\parallel/\perp}}{2}, \qquad A_{\text{OM}}^{\parallel/\perp} = \frac{\sum\limits_{i\in\{\pm 2\delta,\ldots,\pm 7\delta\}} A_i^{\parallel/\perp}}{12}$$

where we have denoted the cross-polarized reference measurements as $\perp$ and the co-polarized measurements as $\parallel$.

Eventually we obtain for the pure HOM-visibility V_{pure} :

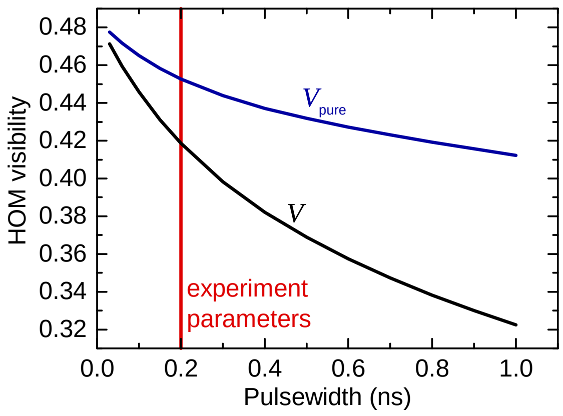

Figure S7. Simulation of pulse width w dependence of HOM visibility with $T_2 = 500\,\mathrm{ps}$, $\Delta - \delta = 0$. Of the three compared parameters, the pulse width w has the least impact on the visibility. A reduction of w by 50% from 200 ps to 100 ps will only reduce the visibility by 3 percentage points.

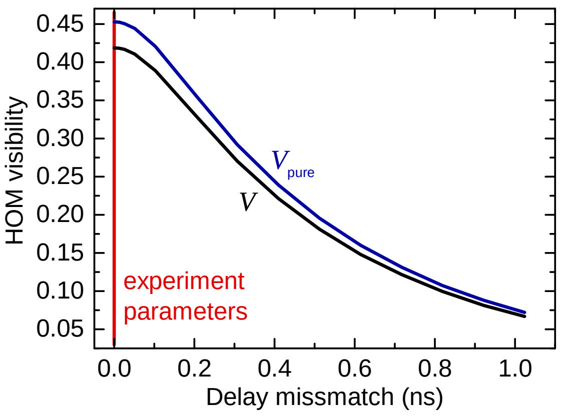

Figure S8. Simulation of delay missmatch $\Delta - \delta$ dependence of HOM visibility with $w = 200\,\mathrm{ps}$, $T_2 = 500\,\mathrm{ps}$. A good matching of the Mach-Zehnder delay to the pulse repetition rate is essential to achieve high visibilities. However our experimental setup was well inside a $|\Delta - \delta| < 20\,\mathrm{ps}$ window.

$$V_{\mathrm{pure}} = (1 - g^{(2)}(0)) \cdot \frac{\frac{A_0^{\perp}}{A_{\mathrm{OM}}^{\perp}} - \frac{A_0^{\parallel}}{A_{\mathrm{OM}}^{\parallel}}}{N} \tag{S5}$$

$$\text{with} \qquad N = 2\left(1 - \frac{A_{\mathrm{NM}}^{\perp} + A_{\mathrm{NM}}^{\parallel}}{A_{\mathrm{OM}}^{\perp} + A_{\mathrm{OM}}^{\parallel}}\right)$$

Based on these considerations we able to determine the pure HOM-visibility corrected for non-ideal multi-photon suppression (see Fig. 5a). Analysing the experimental data presented in Fig. 5b by taking into account Gaussian propagation of uncertainty with the main contribution originating from the shot noise of the peak areas we obtain the pure HOM-Visibility $V_{\mathrm{pure}} = 57(9)\%$.

SVIII. Parameters affecting the Hong-Ou-Mandel visibility

In the following we discuss effects which influence the HOM-visibility and the related photon-indistinguishably in our experimental setting. As mentioned in the main text, the spontaneous lifetime $T_1 = 510\,\mathrm{ps}$, the QD coherence time $T_2 = 500\,\mathrm{ps}$, the laser pulse-width $w = 200\,\mathrm{ps}$ and the laser pulse-area $\int \Omega(t)\mathrm{d}t = 0.9\,\pi$ were determined from experimental data. Furthermore, the delay Δ in the Mach-Zehnder interferometer of the HOM-configuration was optimized to precisely match the pulse distance δ, i.e. $|\Delta - \delta| < 20\,\mathrm{ps}$.

In order to explain the non-ideal HOM visibility and how it can be improved, we calculate the cross-correlogram numerically for different values of the parameters w, Δ and T_2 as described in Sec. SV and determine V and V_{pure} by Eqs. 1 and S5. The results are presented in Figs. S7, S8, S9and S10(only for V), respectively.

The increased pulse-width in our experiment (compared to standard experiments applying excitation pulses with ps pulse-width) has moderate direct influence on the HOM-visibility and leads to a reduction from 47% to 42% for the given experimental parameters. As can be seen in Fig. S8 a delay mismatch would be more detrimental, but this parameter is well under control in experiment with $|\Delta - \delta| < 20\,\mathrm{ps}$. Most problematic is the coherence time which limits the HOM-visibility to values below 50% in the present case (see Fig. S9). Since Fourier-limited linewidths and close to ideal HOM-visibility has been performed for QDs based on the same wafer material we assume that the increase pulse-width leads to enhanced dephasing of the resonantly driven QD exciton, thereby reducing the HOM-visibility. Thus, further optimization need to focus on improved pulse-schemes to improve the $T_2/(2T_1)$ towards the Fourier limit of 1 in order to enhance the HOM-visibility in the applied excitation scheme.

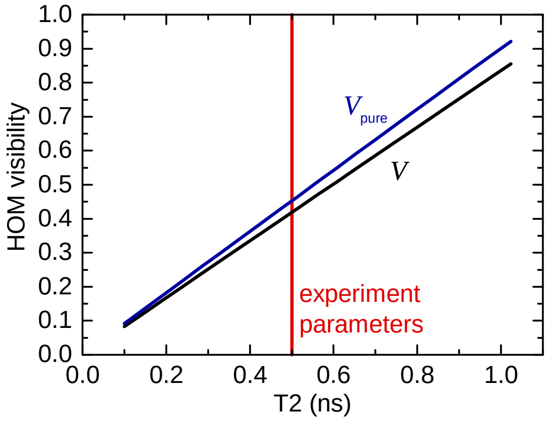

Figure S9. Simulation of QD coherence time T_2 dependence of HOM visibility with $w = 200\,\mathrm{ps}$, $\Delta - \delta = 0$. The QD coherence time has a huge impact: Eventhough the simulations were performed with broad laser pulses, the visibility can exceed $V = 85\%$, $V_{\mathrm{pure}} = 92\%$ in the fourier-limit $T_2 = 2T_1$.

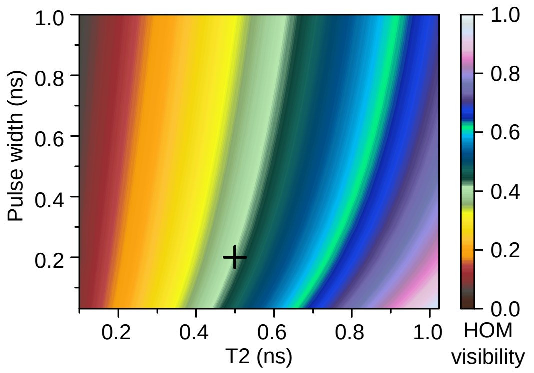

Figure S10. Simulation of both QD coherence time T_2 dependence and pulse width w dependence of HOM visibility with $\Delta - \delta = 0$. The black cross marks the experimental parameters. Obviously a reduction of the pulse width is only significantly beneficial in the almost fourier-limited regime where T_2 is almost equal to $2T_1$.

[1] Gardiner, C. W. & Zoller, P. *Quantum noise: A handbook of Markovian and non-Markovian quantum stochastic methods with applications to quantum optics.* Springer series in synergetics (Springer, Berlin, 2010), third edition edn.

[2] Fischer, K. A., Müller, K., Lagoudakis, K. G. & Vučković, J. Dynamical modeling of pulsed two-photon interference. *New J. Phys.* **18**, 113053 (2016).

[3] Bennett, A. J. *et al.* Indistinguishable photons from a diode. *Appl. Phys. Lett.* **92**, 193503 (2008).

[4] Woolley, M. J., Lang, C., Eichler, C., Wallraff, A. & Blais, A. Signatures of hong–ou–mandel interference at microwave frequencies. *New Journal of Physics* **15**, 105025 (2013).

[5] Flagg, E. B. *et al.* Resonantly driven coherent oscillations in a solid-state quantum emitter. *Nat. Phys.* **5**, 203–207 (2009).

[6] Santori, C., Fattal, D., Vučković, J., Solomon, G. S. & Yamamoto, Y. Indistinguishable photons from a single-photon device. *Nature* **419**, 594–597 (2002).
